# Harnessing spent granular activated carbon from point-of-use water treatment for anaerobic digestion enhancement: characterization and pretreatment effects

**DOI:** 10.1186/s40643-025-00947-9

**Published:** 2025-10-10

**Authors:** J. B. Kemirembe, A. Ayor, T. Kayondo, R. Kayiwa

**Affiliations:** 1 Department of Mechanical Engineering, College of Engineering, Design, Art, and Technology, Makerere University, P.O. BOX 7062, Kampala, Uganda; 2 Chemistry Department, College of Natural Sciences, Makerere University, P.O. BOX 7062, Kampala, Uganda

**Keywords:** Granular spent activated carbon, Biogas, Methane

## Abstract

As an alternative to environmentally unfriendly disposal of spent activated carbon (SAC) from point-of-use water treatment systems, this study harnessed SAC as a co-substrate in anaerobic digestion for biogas production. The study investigated how SAC pre-treatment methods, namely, washing, particle size (milled vs. granular), and dosing levels (2.5%, 5%, and 7.5%), influenced biogas yield, methane content, and retention time using cow dung as the primary substrate. SAC from chlorinated water purification (KC) and the other from borehole water treatment (KZ) were investigated in this study. KZ had a higher %TOC (5.4955%) compared to < LOD for KC, indicating it retained more usable carbon for microbial activity. However, KC had a higher surface area (110.58 m^2^/g) than KZ (78.41 m^2^/g). suggesting better microbial support. Digesters dosed with 2.5% and 5% SAC generally maintained the most stable retention time, sustaining active digestion across the full 30-day period. Overall, unwashed granular KZ dosages of 2.5%, 5.0%, and 7.5% respectively, supported the highest and most stable biogas output at 5047 mL, 2605 mL, and 1685 mL, respectively. A 2.5% KZ dosage outperformed the control (pure cow dung), which produced 2624 mL. Milled washed KC dosages of 2.5%, 5.0%, and 7.5% generally showed lower biogas outputs, yielding 217.8 mL, 540.8 mL, and 300 mL, respectively. Their relatively low performance suggests that even after washing, the milled coconut husk SAC, at these concentrations, did not significantly enhance microbial activity, or perhaps the fine particle size led to aggregation or hindered mass transfer. KZ-dosed digesters’ highest methane composition range was 14.90–37.70% whereas for KZ digesters it was 47.73–52.53% compared to the control’s 33.70%. These findings underscore the complex interplay between particle size, washing, and dosage of SAC for enhancing anaerobic digestion, necessitating optimization.

**Graphical abstract:**

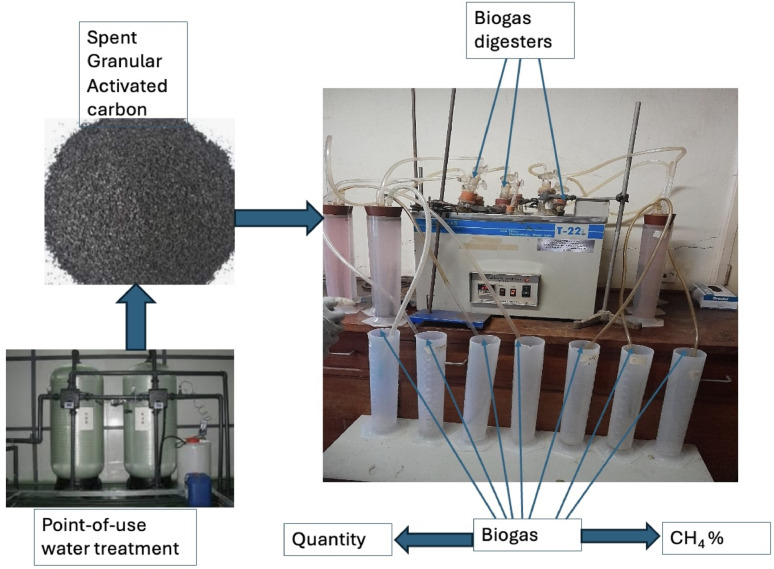

## Introduction

From 2015 to 2021, the global demand for activated carbon (AC) increased from 2.7 to 5.4 million metric tons, and 38% of this demand was driven by its use in wastewater treatment plants to meet environmental sustainability standards (Ho 2022). Synthetic AC adsorbents are preferred for their low production cost, high surface area, and large pore volume, making them effective in removing inorganic pollutants as well as eliminating unpleasant odors in wastewater (Tolkou et al. 2024).

Activated Carbon (AC) is widely used in point-of-use water treatment systems as an adsorbent in relatively smaller amounts compared to the wider-scale wastewater treatment systems. After usage for a certain period, the AC is then called “Spent,” implying it can no longer adsorb contaminants effectively. The disposal of spent activated carbon (SAC) from point-of-use water treatment systems poses a salient environmental threat. These systems utilize AC in relatively small amounts and are typically set up and operated in homes (Menya et al. 2023). They are therefore not given as much attention as the commercial, large-scale water treatment plants regarding the handling of their SAC. The difficulty associated with SAC management has driven most point-of-use water treatment plant operators to opt for disposing of the SAC at domestic waste collection points and, hence, ending up in landfills (Siddiqua et al. 2022). Although it may not be considered a hazard in the short term, its accumulation raises concerns in the long term due to the formation of greenhouse gases, such as methane and carbon dioxide, which affect the environment. Landfilling is often associated with more environmental risks, especially in developing countries where landfills are non-engineered to contain hazardous waste (Ayisi et al. 2022; Idowu et al. 2019). Regeneration is another route for the secondary life of SAC. It restores the adsorption capacity of SAC to a commensurate but lower capacity than that of the primary AC capacity (Leong et al. 2018; Márquez et al. 2022; Yadav et al. 2022). Approximately 68% of granular AC is recoverable, whereas over 95% of powdered AC is disposed of in landfills (Kayiwa et al. 2021). Besides, the cost of regeneration is often as high as the production of AC, making regeneration economically unfeasible. This, therefore, requires processes that can harness such small amounts of SAC feasibly. Anaerobic enhancement has been suggested as a potential upcycling route for such SAC (Kayiwa et al. 2025).

On the other hand, biogas production from organic waste, which for decades has offered a renewable energy resource, still faces shortcomings regarding the system efficiencies, substrate quality, and biogas storage. Biogas systems act as an efficient waste management tool as well as to mitigate harmful methane emissions from organic matter and livestock waste, and to reduce the danger of disposing of fresh organic matter into the soil. The byproducts of the process, particularly organic fertilizer, are valuable for improving soil fertility (Béghin-Tanneau et al. 2019; Cristina et al. 2019).

Despite the environmental and economic benefits, the efficiency of biogas is often constrained by limited microbial activity and process parameters that affect the bacteria at different stages of biogas production, shortening the retention time. Co-digesting the would-be stand-alone substrates with more robust materials like digestate co-substrates and AC has been reported to enhance digester performance (Dennis 2015; Valentin et al. 2023). These co-substrates introduce and or kick-start microbial activity besides providing amicable conditions for microbial proliferation (Al-Iraqi et al. 2023; Johnravindar et al. 2020). Virgin AC has been applied to enhance biogas production in past studies (Liu & Jiang, 2021; F. Wu et al., 2022; Yan et al. 2020). However, in some studies, a reduction in methane yield upon addition of virgin granular activated carbon (GAC) has been reported. For example, Jiang et al., (2021) reported a 6.5%–36.9% reduction in methane yields of raw sludge when granular activated carbon was added due to the non-selective adsorption of the GAC. Such discrepancies, along with the nature of SAC from point-of-use water treatment and prior treatment on biogas quantity and quality, have not been explored.

This study aimed to uncover the effect of dosages, washing, and milling of the granular SAC from coconut husks on the performance parameters of the biogas digesters. The SAC from point-of-use water treatment plants, one handling borehole water from underground and another treating chlorinated water, would provide insights into the inherent adsorbate effects and SAC dosages on digester performance and any treatments needed if either of the SACs is to be deployed as a co-substrate.

## Materials and methods

### Feedstock collection and treatment

In this study, spent activated carbon (SAC) was sourced from a water treatment company that uses coconut shell granular activated carbon at its point-of-use water treatment sites. Samples were collected from two sites: A children’s home in Kyankwanzi district, western Uganda (code-named “KZ”), treating borehole water, and a medical centre in Kampala, Uganda (code-named “KC”), purifying chlorinated tap water. Approximately 1 kg of SAC was collected per site, transported in carbon filter housings, and stored at − 4 °C. Additionally, cow dung was collected from a Wakiso district farm, cleaned of debris, and stored in a plastic bucket. For the experiments, 675 g of SAC was initially washed with 450 mL of distilled water and then twice with 150 mL. Then, the washed SAC was filtered off and milled using a mortar and pestle to about 0.5 mm particle size.

### Characterization of the SAC

#### pH

0.3 g of each of the 12 samples (triplicate for unwashed milled and granular for KC and KZ, respectively) was accurately weighed and placed into a Falcon tube, followed by the addition of 30 mL of distilled water. The tube was then securely closed, and the sample was mixed using a Stuart Scientific Autovortex Mixer SA1 vortexer for approximately 1 min. These quantities were selected based on a 1% weight-to-volume ratio protocol for SAC and distilled water. The pH of the mixture was measured using a pH meter (HQ4300 Hach, Canada) by immersing the probe in the mixture.

#### Nitrogen content

The nitrogen content of SAC and cow dung was determined using the Kjeldahl method. The Kjeldahl method using kjeldahl KDN-05D and ATN-100 was used to determine the nitrogen content in the cow dung and SAC. The process began with the digestion of the 9 samples (triplicates of cow dung, KC, and KZ) of 0.2 g weight using 10 mL of concentrated sulfuric acid (H₂SO₄) in the presence of CuSO_4_ and K_2_SO_4,_ which acted as catalysts facilitating the conversion of nitrogen into ammonium sulfate (NH4)_2_S0_4_ for about eight hours. 10 mL of distilled water was added to dilute the sample, and 70 mL of NaOH was added, converting the ammonium ions into ammonia gas. The released ammonia was then distilled into a boric acid solution with an indicator that is 14 mL of methyl red and 20 mL of ethanol, where it was trapped and subsequently titrated with a standardized acid solution, 0.05 M HCl, until the solution turned back to pale pink. The nitrogen percentage in the sample was then calculated according to Eq. 1.

1 $$\% \text{Nitrogen}=\frac{V\times C\times 0.014}{W}\times 100$$

*where.*

V are the titration values, c is the molarity of hydrochloric acid used to titrate, which is 0.05 M, and w is the weight of the sample.

For computation of the C/N ratio of the substrates in the digesters, the total nitrogen content was derived from,

2 $$ \begin{aligned} {\text{Total Nitrogen content}} = & ({\text{Cow dung }}(g) \times \% N\;{\text{in}}\;{\text{cow}}\;{\text{dung}}) \\ & \quad + ({\text{SAC }}(g) \times \% N\;{\text{in}}\;{\text{SAC}}) \\ \end{aligned} $$

#### Total organic carbon

Nine samples (3 samples for KC and KZ, respectively, and 3 samples for cow dung) were analyzed for total carbon content using a spectrophotometer. Precisely 0.05 g of each sample was weighed using an analytical balance and transferred into separate 100 mL conical flasks. To each flask, 10 mL of 0.167 M potassium dichromate (K₂Cr₂O₇) solution was added. This was followed by the slow addition of 20 mL of concentrated sulfuric acid (H₂SO₄) to initiate the oxidation of organic carbon. The reaction mixtures were then carefully diluted with 80 mL of distilled water to bring the volume up and reduce the acidity. The flasks were allowed to stand undisturbed for 30 min to ensure complete reaction and stabilization of the solution before spectrophotometric analysis. The resultant solutions were poured into a 10 mm glass cell, which was fixed in the spectrophotometer compartment. The absorbance was measured at the appropriate wavelength using a UV–Vis spectrophotometer (Drawell, DV 8200, China), and total carbon concentration was calculated based on a standard calibration curve. The value read from the spectrophotometer is the concentration of the organic carbon that was oxidized from the compounds (mgC/L), which is represented as C oxidized.

3 $$C oxidized(mgC/g)=\frac{C oxidised (mgC/L)\times 0.1L}{W}$$

4 $$\% TOC=\frac{C oxidised(mgC/g)\times 0.77\times 100}{1000}\times \frac{100}{58}$$

where;

W-weight of the sample and C oxidized is the concentration on the photospectrometer in mgC/L.

For the computation of the C/N ratio of the substrates in the digesters, total carbon content was derived from,

5 $$ \begin{aligned} {\text{Total}}C & = \left( {{\text{Cowdung}}\left( g \right) \times \% {\text{TOC}}\;{\text{in cowdung }}} \right) \\ & \quad + \left( {SAC\left( g \right) \times \% {\text{TOC in}}\;{\text{SAC}}} \right) \\ \end{aligned} $$

#### Functional group analysis

The functional groups in the unwashed SAC samples (KZ and KC) were identified using Fourier Transform Infrared (FTIR) spectroscopy (Shimadzu FTIR-8400S, Japan). The samples were first dried at 150 °C for 1 h, milled into a fine powder, and sieved for uniformity using a 0.5 mm sieve. To prepare the FTIR pellets, 0.002 g of SAC was mixed with 0.2 g of potassium bromide (KBr) in a 1:100 ratio. This mixture was pressed into transparent pellets using a hydraulic press at 80 bars. Three pellets were prepared for each sample, and a blank KBr pellet was also made to calibrate the FTIR spectrometer by eliminating background absorbance. The pellets were mounted onto the FTIR spectrometer’s sample holder. During the scan, the infrared light passed through the pellets, and the various functional groups in the SAC samples absorbed light at specific wavelengths. The spectrometer recorded the resulting absorption spectra, which were analyzed to identify functional groups.

#### Porosity and surface area

The iodine number, used to estimate the surface area and porosity of spent activated carbon (SAC), was determined using a standard iodometric method. SAC samples were oven-dried at 150 °C for 1 h, milled to an averagely 0.5 mm particle size. For analysis, six samples (triplicates of KZ and KC of 2.5 g each). 2.5 g of SAC were added to 15 mL of Hanus iodine solution. Titration was performed using 0.1 N sodium thiosulphate (6.5 g in 500 mL distilled water) until the solution turned pale yellow. After that, 2 mL of starch indicator (2 g in 150 mL boiling water) was added. Titration continued until the blue-black colour disappeared. The iodine number was calculated using Eq. 6.

6 $$\text{Iodine number }(\text{mg}/\text{g})=(\frac{{(V}_{b}-{V}_{s })\times N\times 126.93}{m} )$$

where;

V_b_ = Volume of Na₂S₂O₃ used in blank titration in mL, V_s_ = Average volume in sample titration in mL, N = Normality of sodium thiosulfate in eq/L, Molar mass of iodine (g/mol) = 126.93, and m = Mass of activated carbon used (g).

According to Ziemińska & Doczekalska,(2024), the resulting multiplier typically ranges from 0.988 to 1.884. In this study, a value of 1.32 $m/mg$ was selected as it lies near the midpoint of this range. Therefore, the surface area can be estimated by multiplying the iodine number by this factor.

7 $$ {\text{Surface}} {\text{Area }}\left( {m/g} \right) = {\text{Iodine Number}} \left( {mg/g} \right) \times 1.32 $$

### Experimental setup of the anaerobic digesters

**Fig. 1:**
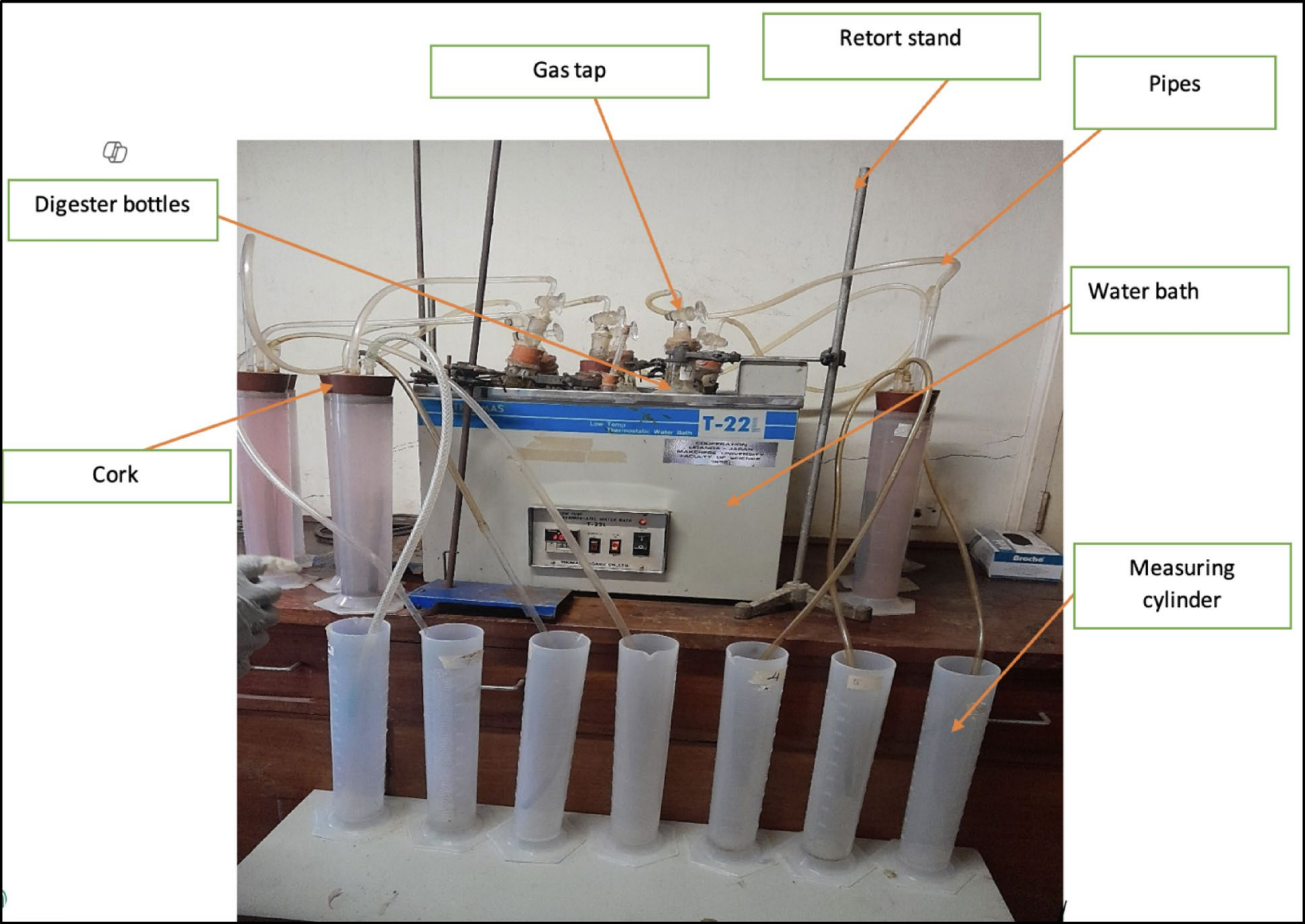
Experimental setup

Anaerobic digestion experiments were conducted using 1000 mL Perspex glass bottles as digesters, which were cleaned with deionized water. Cow dung was mixed with distilled water in a 1:1 weight ratio to form a slurry, with SAC added at 2.5%, 5.0%, and 7.5% (22.5, 45.0, and 67.5 mL, respectively), while the control contained cow dung slurry only. Each digester contained 900 mL of substrate. The digesters were sealed with corks, equipped with gas taps, and placed in water baths suspended on retort stands. The baths maintained mesophilic temperatures (36 to 38 °C) via thermostats and heaters. Biogas was collected through rubber pipes leading to measuring cylinders with 0.01 M HCl, sealed to prevent leakage, and then displaced into cylinders with distilled water to measure gas production using the water displacement method. The digesters were gently shaken to prevent microbial buildup at the top and ensure microbial distribution throughout. The experimental setup is shown in Fig. 1

Digesters 1 to 19 were configured with varying treatments of SAC and cow dung to assess the SAC’s performance as a co-substrate in biogas production. Digester 1 was the control and contained only cow dung with no SAC. Digesters 2 to 4 contained unwashed, milled KC, at 2.5%, 5.0%, and 7.5%, respectively. The remaining digester groups (3 digesters in each group) were dosed with 2.5, 5.0, and 7.5 volume % of the respective SAC in ascending order of the code numbers, as summarized in Table 1. A biogas analyzer (RASI 700 Bio, UK) was used to measure the methane percentage composition in the biogas produced during the digestion process by tapping the gas through the analyzer’s pitot tube until the readings on the display were stable. The digester setups enabled a structured study of how the SAC source (nature of adsorbates), pre-washing, particle size, and SAC dosage influence biogas yield and quality, and retention time in anaerobic digestion.

**Table 1:**
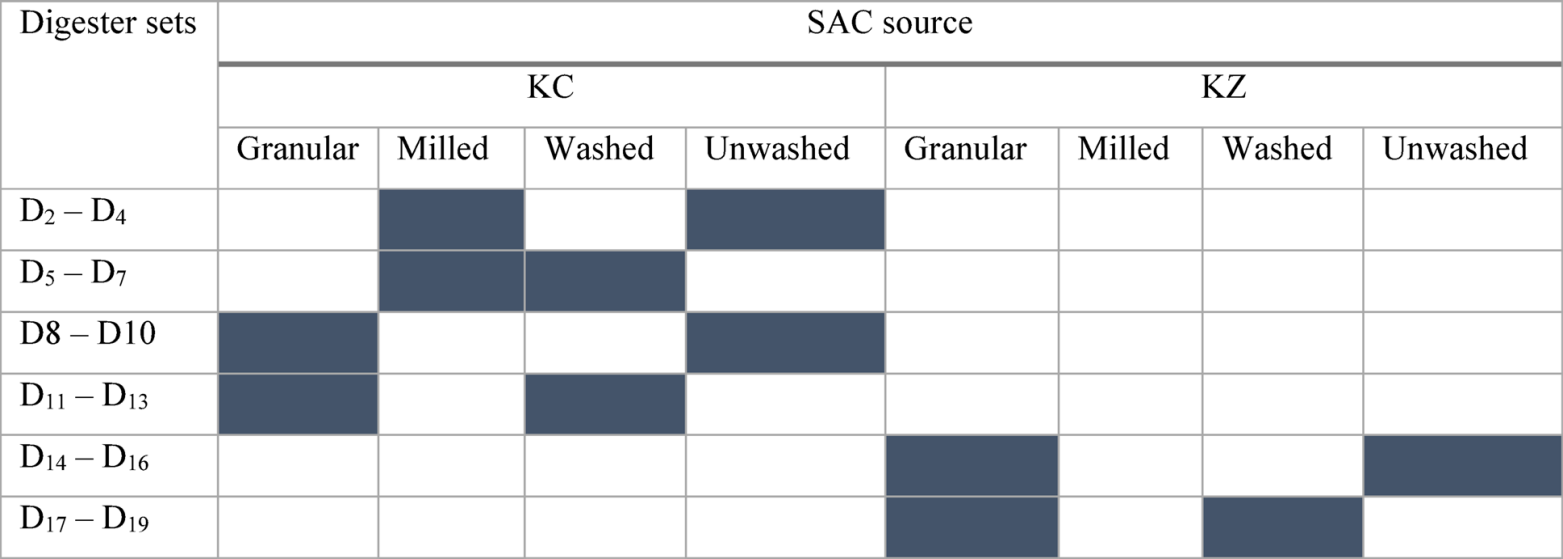
Digester sets with the corresponding SAC form

## Results and discussion

### Characteristics of SAC

#### pH

The pH of the SAC is a critical factor influencing its performance as a co-substrate in anaerobic digestion (AD), particularly due to its effect on microbial activity and biogas yield. Methanogenic bacteria, which play a vital role in methane production, thrive within a pH range of 6.5 to 8.0. The effects of pretreating SAC on pH are presented in Table 2.

**Table 2:** pH values for the unwashed and washed SAC

SAC samples	pH	
	unwashed	washed
KC (granular)	8.57 ± 0.24	8.14 ± 0.21
KC (milled)	7.74 ± 0.14	6.58 ± 0.11
KZ (granular)	8.48 ± 0.25	6.70 ± 0.23
KZ (milled)	6.97 ± 0.02	6.44 ± 0.04

The SAC samples exhibited different pH values depending on the primary water quality the SAC was treating and the particle size (milled and granular) of the SAC. Notably, the granular SAC samples were more alkaline compared to the milled samples. A similar observation was reported by Lee et al. (2023) in their study on engineered ball-milled colloidal activated carbon material for the advanced oxidation process of ibuprofen. This difference can be attributed to the increased exposure of acidic functional groups, such as carboxyl and phenolic groups, on the carbon surface during milling. Although milling does not alter the chemical composition of SAC, it enhances the exposure of internal surfaces and reactive sites, consequently lowering the overall pH. These observations align with the findings of Abulikemu et al. (2023). They indicate that surface modification through milling can impact the microbial environment and influence digestion dynamics.

#### Surface area and porosity

The SAC yielded iodine numbers of 83.77 mg/g and 59.40 mg/g for KC and KZ, corresponding to surface areas of 110.58 m^2^/g and 78.41 m^2^/g, respectively as shown in Table 3. These values are considerably lower than those typically found in fresh activated carbon used in water treatment, which often exceed 450 m^2^/g (Dong et al. 2018).

**Table 3:** Iodine number and surface area for the granular SAC

	KC	KZ
Iodine number $(mg/g)$	83.774 ± 0.02	59.403 ± 0.01
Surface area $(m/g)$	110.581 ± 0.04	78.412 ± 0.01

This reduction suggests degradation of the surface structure due to prolonged use in water treatment systems. The extended exposure to contaminants over time likely contributed to the loss of porosity and surface area, further indicating that the SAC was well-utilized before being repurposed for this study. The surface area and porosity of SAC are vital in supporting microbial growth and enhancing substrate-microbe interaction. Higher values improve microbial attachment and digestion efficiency, potentially leading to increased biogas production (Li et al. 2022). Nevertheless, even with reduced surface area, the SAC remains viable as a microbial support medium in AD. Unlike its role in water purification, where a high surface area is essential for adsorption, in biogas systems, its function is to enhance microbial activity. Therefore, the remaining surface structure was still adequate to promote microbial attachment and support digestion efficiency.

#### Carbon and nitrogen content

The carbon and nitrogen content of SAC directly influences the C/N ratio, which is crucial for balanced microbial activity during anaerobic digestion. Adequate carbon provides energy, while nitrogen supports cell growth. There were variations in carbon and nitrogen levels among the SAC samples that could have affected the digestion environment and likely contributed to differences in biogas yield. Table 4 shows the variations in % nitrogen and % carbon in each SAC sample, providing insight into the potential nutrient availability and biological activity.

**Table 4:** Nitrogen and Carbon percentage compositions in the individual SACs and cow dung

	KZ	KC	CW
% Nitrogen	0.390 ± 0.0494	0.5147 ± 0.0432	1.742 ± 0.0629
Mass in mg/g of Nitrogen	3.90 ± 0.494	5.147 ± 0.432	17.42 ± 0.629
% TOC	9.13 ± 0.651	< LOD	29.25 ± 1.1038
Mass in mg/g of Carbon	91.3 ± 6.51	< LOD	292.5 ± 11.038

LOD = limit of detection

The total organic carbon wasn’t detected in KC because KC SAC was treating chlorinated water, which could probably have contained no or relatively few microorganisms responsible for carbon breakdown. The carbon-to-nitrogen (C/N) ratios across all digesters ranged from 16.01 to 17.02, as shown in Table 5. The cow dung exhibited higher-than-expected nitrogen levels, due to the animals being fed on silage, a protein-rich feed that increases nitrogen content in manure. SAC, particularly KZ, contributed slightly more carbon than KC, resulting in marginally better C/N ratios in those digesters.

**Table 5:** Digester C and N contents with varying SAC source and dosages

SAC source	Digester	TOC (mg)	N (mg)	C/N
Control (cow dung only)	D1	131.63	7.84	16.79
KC	D2	128.32	7.76	16.54
	D3	125.01	7.68	16.28
	D4	121.70	7.60	16.01
	D5	128.32	7.76	16.54
	D6	125.01	7.68	16.28
	D7	121.70	7.60	16.01
	D8	128.32	7.76	16.54
	D9	125.01	7.68	16.28
	D10	121.70	7.60	16.01
	D11	128.32	7.76	16.54
	D12	125.01	7.68	16.28
	D13	121.70	7.60	16.01
KZ	D14	130.37	7.73	16.86
	D15	129.11	7.62	16.94
	D16	127.86	7.51	17.02
	D17	130.37	7.73	16.86
	D18	129.11	7.62	16.94
	D19	127.86	7.51	17.02

Although these values fall below the C/N range of 20:1 to 30:1 recommended for optimal performance of anaerobic digesters, the digesters maintained stable performance, probably due to less ammonia inhibition and microbial imbalance. The performance of these digesters, despite low C/N ratios throughout the experiment, indicates that SAC, though not a contributor of high carbon content, helped stabilize the digestion environment when paired with nitrogen-rich cow dung. These findings support the idea that while the 20–30 range of C:N ratio may be commendable for methane production, successful digestion is still achievable outside that window under balanced conditions.

#### Functional groups

**Fig. 2:**
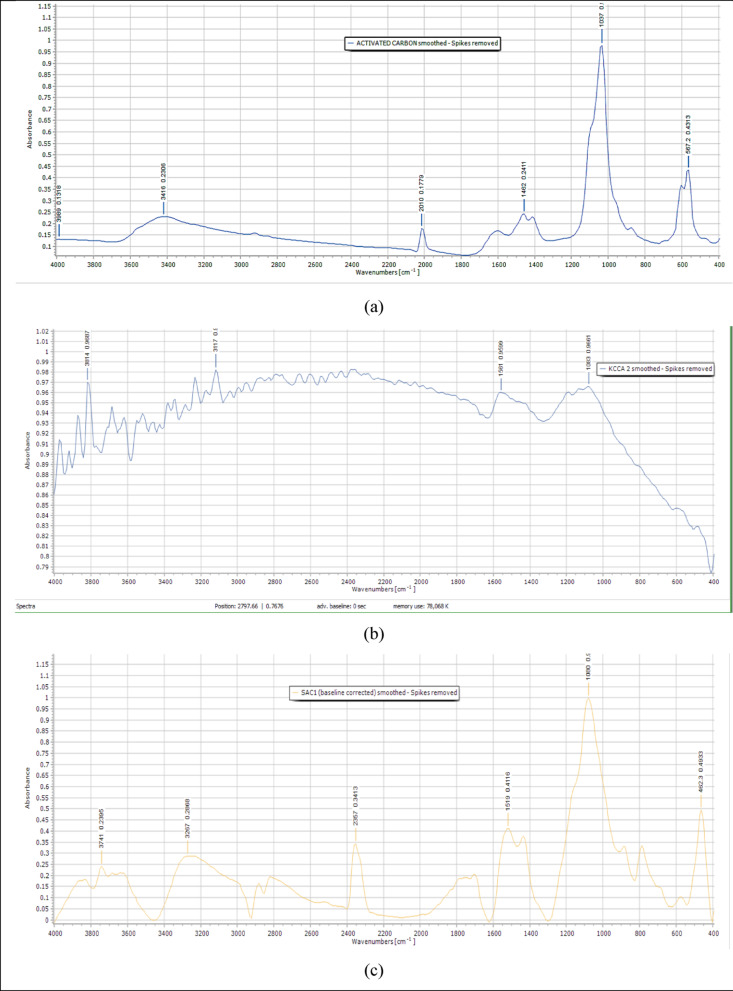
FTIR spectroscopy for the virgin AC (**a**), KC (**b**), and KZ (**c**)

The FTIR analysis identified functional groups and their intensities on the SAC surface, which could influence adsorption capacity and microbial interaction. The presence of oxygen-containing groups (like hydroxyl and carboxyl) may enhance microbial attachment and activity, hence affecting biogas performance. Figure 2a shows the FTIR data of the original activated carbon that was used in this study as a reference. The spectrum highlights the original functional group profile before exposure to water treatment processes.

Figure 2b shows the FTIR spectrum of KC with several notable changes in surface chemistry compared to the virgin carbon. Increased intensities in several oxygen-containing functional groups compared to virgin activated carbon were observed. The hydroxyl group (3400–3600 cm⁻^1^) rose to 0.9237, indicating surface oxidation likely caused by contact with chlorine and other oxidants during water treatment. The carbonyl group (1700–1720 cm⁻^1^) also increased to 0.9485, further confirming chemical modification. In the carboxylate region (C–O–O, 1380–1270 cm⁻^1^), intensity increased to 0.9348, suggesting enhanced polarity and cation-exchange capacity.

Additionally, the aromatic graphitic band (1600–1620 cm⁻^1^) increased to 0.9397, indicating a more ordered carbon structure favorable for microbial adhesion. The Aromatic C–H region (800–900 cm⁻^1^) also showed an increase in intensity to 0.8971, suggesting that the SAC surface retained hydrogen-bearing aromatic structures, which may support additional microbial interactions and contribute to structural stability.

Figure 2c shows FTIR data for KZ, which was obtained from a system treating borehole water. It exhibited much lower intensities in these functional regions. The hydroxyl band decreased to 0.05445, and the carbonyl group was only moderately increased at 0.1988. The COO band dropped to 0.0038, and the aromatic graphitic band declined to 0.0054, implying that organic contaminants in untreated borehole water may have blocked active sites or caused fouling. The Aromatic C–H band, however, slightly increased to 0.2523, indicating that some aromatic integrity was retained.

The FTIR analysis revealed that the KC, which was used to treat chlorinated tap water, underwent noticeable surface changes, gaining more hydroxyl, carbonyl, carboxylate, and aromatic structures. These changes suggest that KC became more chemically active (Fang et al. 2021) and better suited for supporting microbial life, which is beneficial for biogas production. In contrast, KZ used to treat underground borehole water showed less surface oxidation and a reduction in key functional groups. This implies less reactivity and potentially less effectiveness in supporting microbial activity needed for efficient biogas generation. This could probably have been due to the water quality. Overall, the water quality influences how the carbon surface groups evolved, and this directly affects the SAC performance as a bio-co-substrate in biogas systems.

### Effect of the SAC on pH and retention time

#### Effect on the substrate pH

Table 6 presents the initial and final pH and temperature values recorded for each digester. These parameters are critical indicators of microbial activity and process stability during anaerobic digestion.

**Table 6:** Initial and final pH and temperatures for the digesters

Digester	Initial pH	Initial temperature ($^\circ{\rm C} )$	Final pH	Final temperature ($^\circ{\rm C} )$
D1	6.81	25.4	6.64	31.7
D2	6.92	25.2	5.52	30.2
D3	6.91	25.2	5.52	30.6
D4	6.95	25.1	5.85	32.4
D5	6.96	25.4	5.68	31.5
D6	6.95	25.4	5.58	30.6
D7	6.92	25.4	5.67	31.9
D8	6.83	25.4	6.78	31.2
D9	6.89	25.4	5.93	30.3
D10	6.83	25.4	6.66	31.1
D11	6.79	25.7	6.59	30.0
D12	6.76	25.7	5.97	31.7
D13	6.74	25.7	5.87	29.4
D14	7.54	26.8 $^\circ{\rm C}$	7.12	30.2
D15	7.44	27.0 $^\circ{\rm C}$	6.22	30.5
D16	7.26	26.9 $^\circ{\rm C}$	6.82	31.2
D17	7.57	26.8 $^\circ{\rm C}$	6.64	30.8
D18	7.23	26.9 $^\circ{\rm C}$	6.43	31.1
D19	7.49	27.3 $^\circ{\rm C}$	6.52	30.6

At the start of the experiment, most digesters exhibited an initial pH in the slightly acidic to neutral range (6.74–7.57), which is generally favorable for initiating anaerobic digestion. However, by the end of the retention period, a noticeable reduction in pH was observed in several treatments, particularly those with milled KC without washing (like digesters 2 and 3 dropped to as low as 5.52). This pH decline suggests increased acid production during the hydrolysis and acidogenesis stages, possibly due to rapid breakdown of organics without sufficient buffering. In contrast, digesters with granular SAC (especially unwashed KC, such as digester 8) showed minimal pH change, indicating better pH stability, which may support more balanced microbial activity. These results highlight the importance of SAC form and pre-washing in maintaining optimal pH for biogas production.

#### Overall effect of SAC on retention time

For this study, retention time is defined as the duration over which digestion remained active, producing biogas. The Control**,** DI**,** showed a relatively higher earlier gas production compared to other digesters. However, this was followed by a decline in gas production on day 3 and further to day 4, and several SAC-dosed digesters outperformed it initially, as shown in Figs. 4 and 5. Co-digesting cow dung with SAC enhanced anaerobic digestion by providing a porous surface that supported microbial colonization and stabilized methanogen communities. SAC improved contact between microbes and substrates, adsorbed inhibitory by-products, and gradually released nutrients, sustaining favorable conditions for biogas production. This synergy enabled the recovery of digesters after initial dips.

#### Effect of washing and milling on retention time

**Fig. 3:**
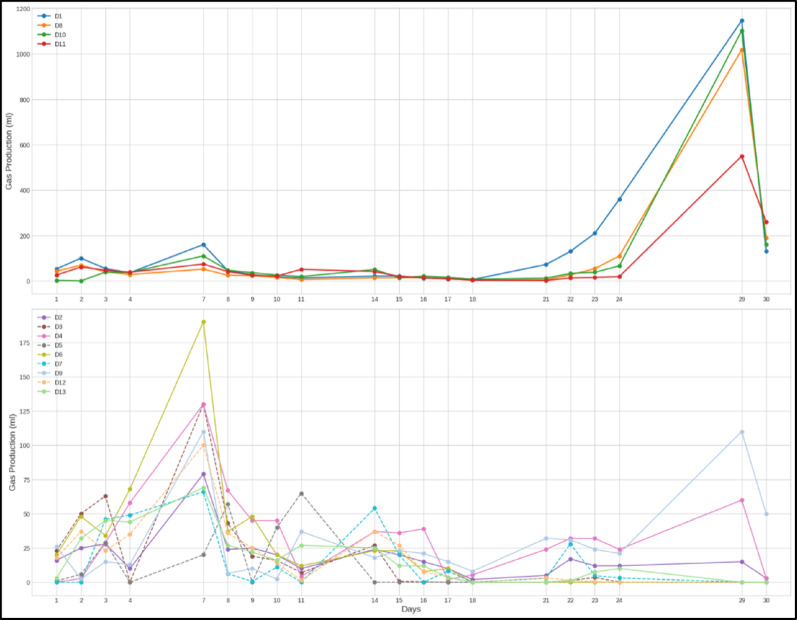
Daily biogas output over the 30 days for KC-dosed digesters

All digesters, including the control (D1, cow dung only), showed an initial peak in biogas production followed by a decline and then recovery, sustaining gas production to varying extents as shown in Fig. 3. Digesters with unwashed, milled KC (D2&D4) displayed a longer retention, maintaining biogas production over time despite an initial dip, which was followed by recovery and sustained output. Unwashed, granular SAC (D8–D10) also improved retention over the control as well as the milled unwashed. Digesters with washed SAC, both milled (D5–D7) and granular (D12&D13), showed relatively shorter retention, with weaker recovery after the initial decline, performing slightly better than the control but worse than unwashed SAC.

The retention patterns reflect the synergistic effects of washing and milling. Unwashed SAC probably retained adsorbed organics and biofilms from prior use, gradually releasing degradable matter and supporting microbial communities, which allowed digestion to recover and continue after early substrate depletion (Wu et al. 2021). Milling further amplified this by increasing surface area, vital surface groups, and microbial accessibility, resulting in the strongest recovery and longest retention in D2&D4. Fang et al., (2021) also reported a significant improvement in the density of the surface functional groups of activated carbon via high-intensity grinding. Granular, unwashed SAC (D8–D10) also extended retention effectively up to day 30. Washing, however, removed beneficial adsorbates, reducing the ability to sustain microbial activity, as seen in D5–D7 (milled) and D11–D13 (granular), which both showed modest improvements over the control but failed to match unwashed KC performance. This highlights the importance of preserving surface organics in the SAC.

**Fig. 4:**
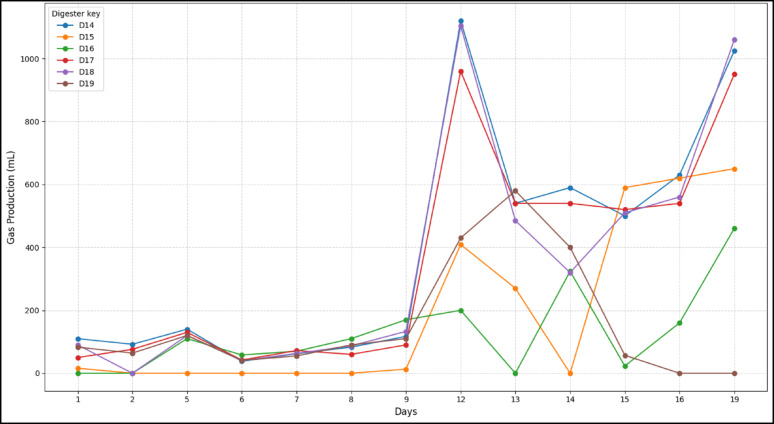
Daily biogas output over the 19 days for KZ-dosed digesters

Notably, the ability of KZ digesters D14 to D19 in Fig. 4 to recover from dips to outputs higher than the initial is an indicator that the granular activated carbon could also enhance recovery and extend retention in case of any external interferences that potentially destabilize the digesters (Kalantzis et al. 2023).

### SAC dosage versus biogas quality and quantity

#### KC effect on biogas quantity

**Fig. 5:**
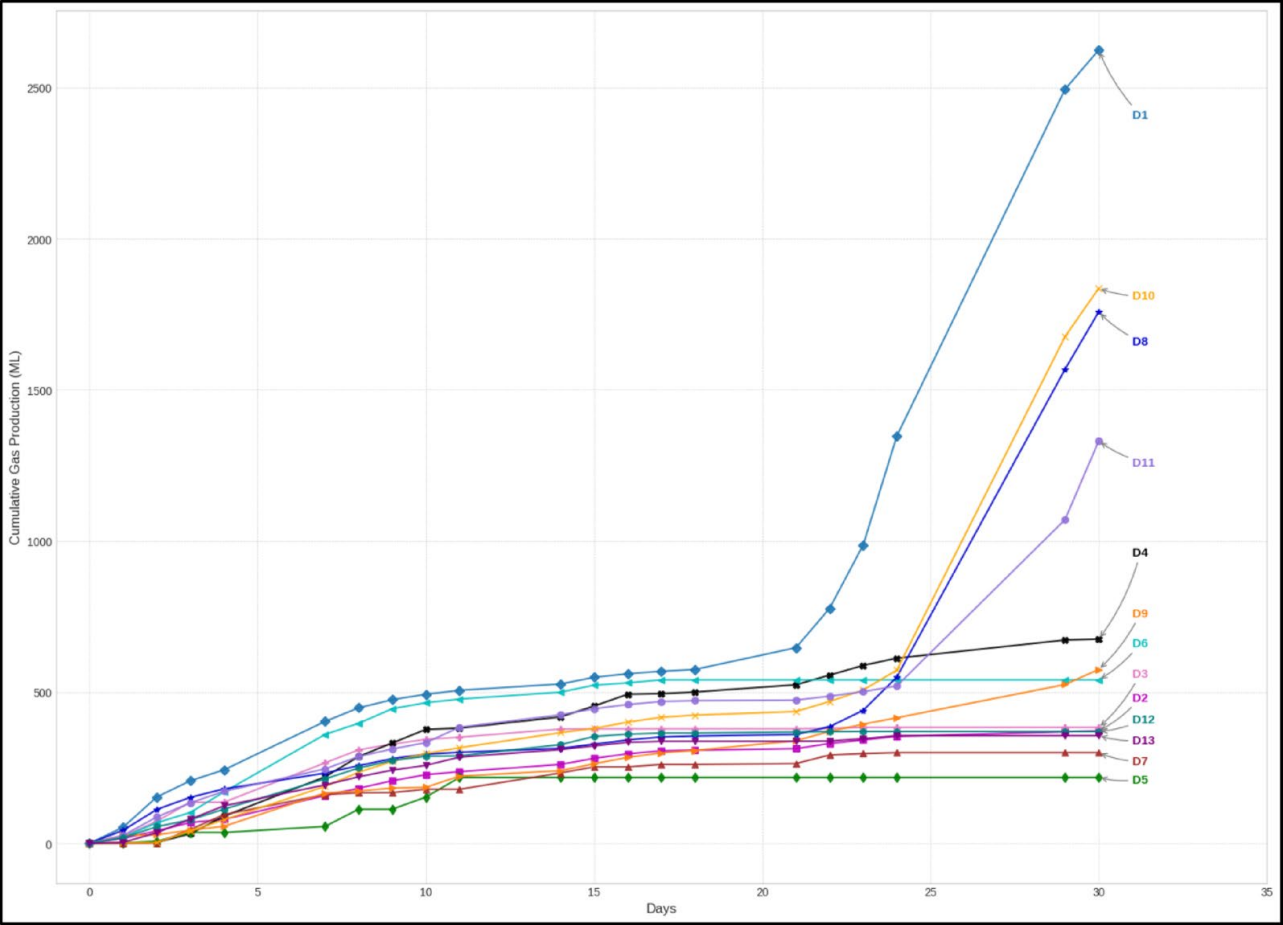
Cumulative gas production for KC-dosed digesters

Figure 5 vividly illustrates distinct cumulative biogas production profiles across digesters D1-D13 over 30 days. D1, the pure cow dung control, achieved the highest yield at approximately 2624 mL, demonstrating robust and sustained production. Among KC-dosed digesters, D10, D8, and D11 showed notably superior performance at 1836, 1757.9, and 1330.6 mL, respectively. In contrast, most other digesters (D2, D3, D4, D5, D6, D7, D9, D12, D13) displayed relatively lower outputs, typically plateauing between 200 and 700 mL.

D1’s robust performance serves as the expected baseline for well-managed anaerobic digestion of cattle manure. Digesters D2, D3, and D4, dosed with unwashed, milled KC at 2.5%, 5.0%, and 7.5%, respectively, showed poor biogas yields of 372, 383.6, and 675.4 mL, respectively. This severe inhibition is likely due to leachable impurities such as heavy metals or recalcitrant polycyclic aromatic hydrocarbons that cannot be removed by chlorination (Kayiwa et al. 2022) and hence were inherent in the unwashed activated carbon. Milling further increases the surface area, potentially exacerbating the leaching of these inhibitors.

Digesters D5, D6, and D7, which contained milled washed KC at 2.5%, 5.0%, and 7.5% respectively, generally showed lower biogas outputs, yielding 217.8 mL (D5), 540.8 mL (D6), and 300 mL (D7). This was somewhat unexpected given that washing typically removes inhibitors. Their relatively low performance might suggest that even after washing, the milled coconut husk SAC, at these concentrations, did not significantly enhance microbial activity, or perhaps the fine particle size led to aggregation or hindered mass transfer (Singh et al. 2020; Xie et al. 2022).

Crucially, digesters D8, D9, and D10, which contained granular unwashed KC (at 2.5%, 5.0%, and 7.5% respectively), exhibited a surprising performance as already highlighted. The high performance of D8 and D10, despite using unwashed material, suggests that the granular form might have played a crucial role. Larger particle size might reduce the leaching of inhibitors compared to milled forms, and the porous structure could still provide sufficient microbial attachment sites and buffering capacity (Nie et al. 2024). This highlights the complex interplay between particle size, washing, and dosage.

The performance of D11, D12, and D13 (granular washed KC) further illustrates the dose-dependency of SAC’s benefits. D11 (2.5%) performed strongly at 1330.6 mL, suggesting an optimal dosage. However, the decline observed in D12 (346.8 mL) and D13 (356 mL) at higher KC dosages indicates that even beneficial SAC can become inhibitory at supra-optimal levels. This could be due to excessive adsorption of essential nutrients required by microbes, physical hindrance to mass transfer, or shifts in microbial community structure (Chiappero et al. 2022), emphasizing the critical need for optimizing SAC dosage for maximum biogas yield. Moreover, Johnravindar et al. (2020) reported that 1.5% (g/g) granular activated carbon increased biogas production and the methane percentage composition to 45% when co-digested with brewery waste activated sludge and food waste.

#### KC effect on the biogas quality

**Fig. 6:**
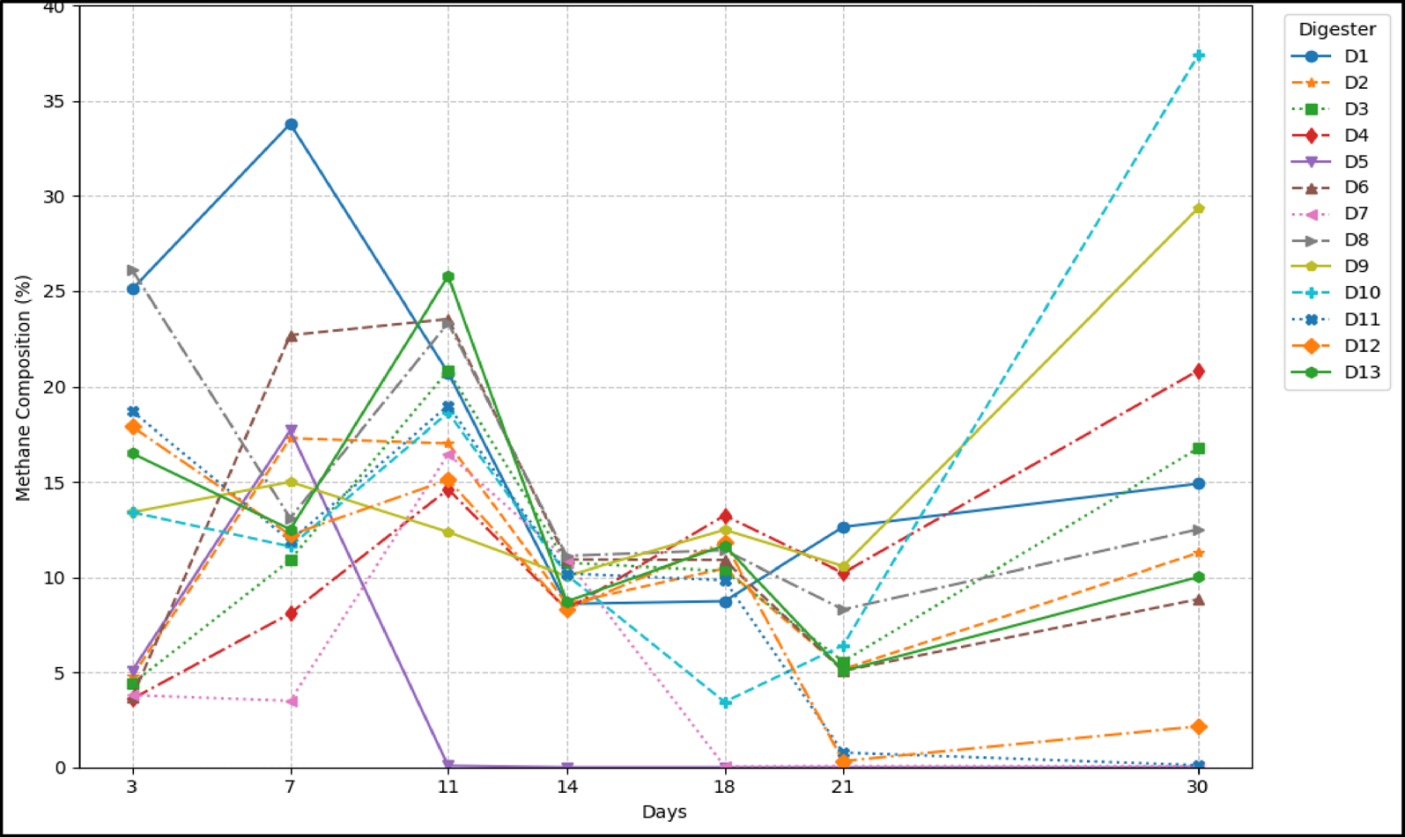
Methane percentage compositions

Generally, the methane content peaked early before fluctuating. Figure 6 illustrates the dynamic changes in methane percentage composition across digesters D1-D13 over 30 days. D1, the cow dung control, showed robust initial methane, peaking at 33.8% on day 7, then stabilizing at 14.9%, setting a baseline for healthy digestion. Digesters D2-D4 (unwashed, milled KC) consistently exhibited very low methane, often near 0%, indicating severe inhibition from leachable impurities inherent in unwashed carbon, exacerbated by milling’s increased surface area (Shekhar Bose et al. 2021). Similarly, D5-D7 (milled, washed KC) also performed poorly, with methane dropping near 0% after day 14. Despite washing, the fine-milled particles might have caused nutrient adsorption or methanogenesis disruption. KC, as per the FTIR analysis, exhibited an intense aromatic hydrocarbon group. Excessive aromaticity tends to render degradable carbon surfaces biologically inert and hence inhibits the growth of methanogens (Florentino et al. 2019; Prem et al. 2023).

D8-D10 (granular, unwashed SAC) showed unexpectedly high methane, with D10 surpassing the control, peaking at 37.7%. The granular form likely limited inhibitor leaching, providing a more stable environment for microbes and attachment sites, surprisingly outperforming some washed counterparts (de Sousa e Silva et al. 2025). D11-D13 (granular, washed SAC) demonstrated dose-dependent behavior. D11 (2.5%) recovered significantly after an initial dip, suggesting optimal conditions. However, D12 and D13 (higher doses) maintained very low methane, near 0%, emphasizing that overdosing could lead to inhibitory effects.

#### KZ effect on biogas quantity

**Fig. 7:**
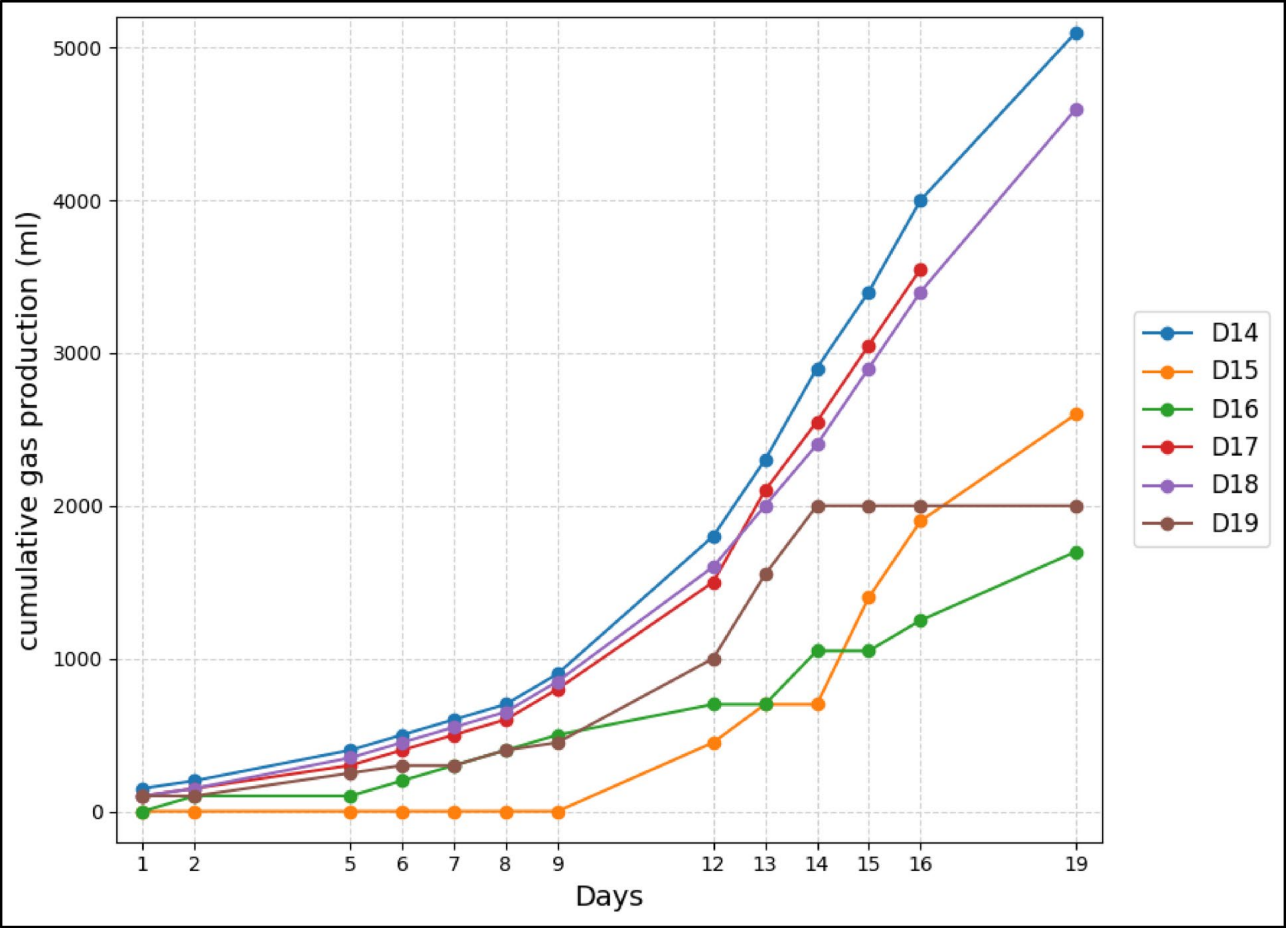
Cumulative gas production for KZ-dosed digesters

The cumulative biogas production trends for digesters D14–D19 reveal marked variations attributed to SAC form and treatment as shown in Fig. 7. The KZ digesters were terminated on day 19 because of signs of reduced biogas production, and continuing beyond this point was unlikely to yield additional trends relevant to the study. D14 achieved the highest yield at 5047 mL by day 19**.** D15 and D16 also increased steadily but reached lower final volumes (2605 mL and 1685 mL, respectively), suggesting that higher SAC doses (in D15, D16) might have inhibited microbial activity or reduced substrate availability.

In comparison, digesters D17–D19 also produced biogas at consistently lower levels. D18 performed best among the washed group at 4610 mL, followed by D17 at 3620 mL, while D19 plateaued early at 2030 mL. This suggests washing removed residual organic carbon and nutrients beneficial for methanogens, reducing biogas potential (Chen et al. 2023; Chiappero et al. 2021).

Overall, unwashed granular SAC (particularly at optimal dosage in D14) supported the highest and most stable biogas output, likely due to its preserved porosity, carbon content, and microbial support (Chen et al. 2023). In addition, the granular unwashed activated carbon could have inhibited the formation of ammonia that would stress the digester system as reported by Xiao et al., (2021). Excessive KZ (D15, D16, D19) or washing (D17–D19) diminished performance, emphasizing the need to balance SAC dose and treatment.

#### KZ effect on biogas quality

**Fig. 8:**
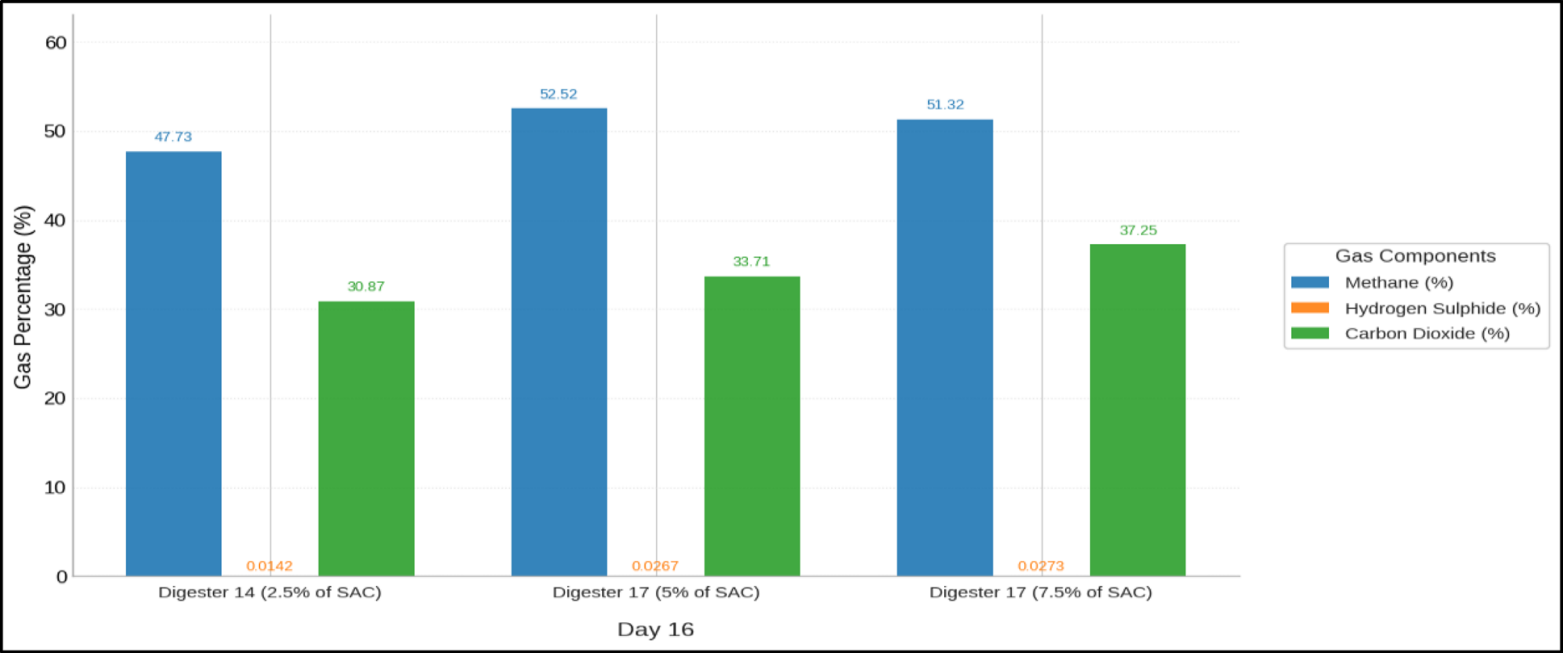
Methane percentage of digesters on day 16

Figure 8 shows the methane percentage for the digesters dosed with KZ on day 16 of the experimental run. Day 16 was selected for gas composition because it coincided with peak biogas production in most of the digesters, allowing us to assess whether methane content followed the same trend as gas quantity. KZ enhanced biogas yield and methane quality, and the performance was closely tied to KZ dosage and presence of organics. Digesters with 2.5% SAC delivered the highest methane percentage composition, with washed KZ (Digester 17) producing 4570 mL and 52.53% methane, while unwashed KZ (Digester 14) performed cumulatively highest at 5047 mL but with 47.73% of methane. These improvements are evidenced by an increase in the C/N ratio from 16.86 to 17.02 and a sufficient surface area (78.4 m^2^/g), which likely helped stabilize pH, buffer inhibitors, and enhance microbial activity (Richard et al. 2019).

Moreover, FTIR spectroscopy analysis revealed that key functional groups, such as carboxyl and hydroxyl, were preserved even after washing, which improved adsorptive interactions. In contrast, unwashed KZ showed evidence of surface fouling, particularly at higher doses, likely obstructing active sites. H₂S levels were significantly reduced in low-dose digesters, reaching as low as 0.0142% in Digester 14, underscoring KZ’s role in scavenging sulphides and enhancing gas purity. Additionally, granular AC has been reported to enrich methanogens capable of direct interspecies electron transfer, which in turn enhances the electron exchange between syntrophs and methanogens to accelerate substrate consumption and methane production (Yang et al. 2017).

Performance declined at higher KZ dosages (5–7.5%), particularly with unwashed KZ. In Digester 16 (7.5% unwashed), biogas dropped to 1685 mL, suggesting nutrient sequestration or substrate over-adsorption (Park et al. 2020). Even with prior washing, overdosing KZ in digesters could reduce the total biogas output, as with Digester 19’s overall output being relatively lower than expected at 2030 mL.

## Conclusions

Characteristically, KZ had a higher %TOC (5.4955%) compared to < LOD for KC, indicating it retained more usable carbon for microbial activity. However, KC had a higher surface area (110.58 m^2^/g) than KZ (78.41 m^2^/g), suggesting better microbial support. Milling reduced the pH but also damaged structural integrity. Overall, granular SAC from either source offered a better balance; however, KZ’s higher TOC and moderate nitrogen content (0.39%) made it more biologically supportive, despite its lower surface area. The reduced microbial support expected in SACs due to reduced surface area can be offset by the presence organic carbon in the SAC.

Digesters dosed with 2.5% and 5% SAC generally maintained the most stable retention time, sustaining active digestion across the full 30-day period. However, some digesters with 7.5% granular, unwashed SAC, maintained commendable gas activity for the full duration, demonstrating that higher SAC dosage can still support full retention when the material is left unprocessed. These results highlight that retention time is influenced not only by dosage, but also by the SAC source and treatment, and that achieving stable, long-lasting digestion depends on finding the right balance between these factors. Further studies on the effect of SAC from various sources on microbial regimes could help uncover the optimal dosages and timing best for introducing SAC in anaerobic digesters.

SAC, especially unwashed and milled, supports longer microbial activity but can initially dilute methane due to increased CO₂ from retained organics on SAC and slower stabilization. While milling can adjust certain properties, preserving structural integrity and maintaining conditions favorable for methanogens are crucial. Selecting the right SAC form ensures improved digestion performance and stability. This complex interplay across particle size, washing, and dosage of SAC for anaerobic digestion enhancement, therefore, requires further optimization studies.

## Data Availability

The data and the materials are all available in this article as well as the supporting information.

## Abbreviations

AC: Activated Carbon
SAC: Spent Activated Carbon
TOC: Total Organic Carbon
